# Connectivity alterations of mesostriatal pathways in first episode psychosis

**DOI:** 10.1038/s41537-023-00339-y

**Published:** 2023-03-14

**Authors:** Nicholas Mark Edward Alexander Hayward, Ana María Triana, Jonatan M. Panula, Tuula Kieseppä, Jaana Suvisaari, Tuukka T. Raij

**Affiliations:** 1 Department of Neuroscience and Biomedical Engineering, Aalto University, P.O. Box 11000, Espoo, FI-00076 Finland; 2 Department of Computer Science, Aalto University, P.O. Box 15400, Espoo, FI-00076 Finland; 3 Department of Clients and Services, Unit of Services, Ministry of Social Affairs and Health, P.O. Box 33, Helsinki, FI-00023 Finland; 4 Finnish Institute for Health and Welfare, Mental Health Team, P.O. Box 30, Helsinki, FI-00271 Finland; 5 Department of Psychiatry, Helsinki University and Helsinki University Hospital, P.O. Box 22, University of Helsinki, Helsinki, FI-00014 Finland

**Keywords:** Psychosis, Psychosis

## Abstract

**Background and hypothesis:**

Pathogenic understanding of the psychotic disorders converges on regulation of dopaminergic signaling in mesostriatocortical pathways. Functional connectivity of the mesostriatal pathways may inform us of the neuronal networks involved.

**Study design:**

This longitudinal study of first episode psychosis (FEP) (49 patients, 43 controls) employed seed-based functional connectivity analyses of fMRI data collected during a naturalistic movie stimulus.

**Study results:**

We identified hypoconnectivity of the dorsal striatum with the midbrain, associated with antipsychotic medication dose in FEP, in comparison with the healthy control group. The midbrain regions that showed hypoconnectivity with the dorsal striatum also showed hypoconnectivity with cerebellar regions suggested to be involved in regulation of the mesostriatocortical dopaminergic pathways. None of the baseline hypoconnectivity detected was seen at follow-up.

**Conclusions:**

These findings extend earlier resting state findings on mesostriatal connectivity in psychotic disorders and highlight the potential for cerebellar regulation of the mesostriatocortical pathways as a target of treatment trials.

## Introduction

While the pathogenesis of first episode psychosis (FEP) remains poorly understood, converging lines of evidence support the dopamine hypothesis, suggesting mesostriatal dopaminergic pathways to be a final common pathway in psychosis^1,2^. The functional anatomy and neurochemistry of dopaminergic pathways, based on animal and human research, are becoming established as the substrate of signal within neuroimaging research (reviewed by McCutcheon et al.^2^). For example, mesostriatal dopamine synthesis increase has repeatedly been observed in positron emission tomography (PET) schizophrenia studies^3^, while dopaminergic drugs induce psychotic symptoms^4^, and all known antipsychotic medications block dopaminergic activity in the striatum^5^. Furthermore, functional striatal abnormalities can already form the basis of predictive markers of schizophrenia^6^.

However, only a small proportion of known risk factors for psychotic disorders is related directly to dopaminergic pathways. Also, multiple psychosis risk factors and related brain findings point to complex alterations in the brain’s neuronal networks that may contribute to dysregulation of the mesostriatal pathways. One option to assess such up-stream alteration is to study functional connectivity of the striatum and especially the midbrain components of the mesostriatal pathways^7^. Previous fMRI studies have focused on mesolimbic and mesostriatal functional connectivity^2^, but much less is known about extra-striatal midbrain functional connectivity in psychotic disorders^7,8^. In one study using a probabilistic ventral tegmental area (VTA) template seed, Nakamura and colleagues found no group differences in resting state connectivity of the ventral tegmental area between patients with schizophrenia and healthy control subjects^7^. However, resting state VTA hypoconnectivity with the cerebral cortex is associated with avolition in schizophrenia subjects^8^. Overall, midbrain functional connectivity and its associations with psychosis symptoms warrant further research.

The midbrain components of the mesostriatocortical pathways are spatially restricted and may be difficult to define based on atlases. Therefore, we used an alternative functional localization approach based on functional connectivity alterations with the striatum. The dorsal striatum is often termed ‘associative’ as it is involved in higher cognitive functions through cortical projections^9^ and may show the largest dopaminergic dysfunction in schizophrenia^1^. Conversely, the ventral striatum is associated with aberrant mesolimbic connectivity linked to positive symptoms (hallucinations and delusions) in schizophrenia^10^ and those at high risk of psychosis^11^. Recent research has also strongly connected the ventral striatum with reward deficits^12^.

Our fMRI seed selection approach intends to create seed regions defined by striatal functional activity rather than only its anatomical borders. To do this, seeds were based on voxels linked to ‘associative’ (dorsal) and ‘motivational’ (ventral) striatal functions in research published in the NeuroSynth database (neurosynth.org). Then the midbrain seeds for further connectivity analyses were defined as voxels in the midbrain showing a group difference between FEP patients and control subjects in functional connectivity with either of the striatal seeds. In addition to seed selection, we aimed to complement earlier literature by using a rare longitudinal sample of first-episode psychosis (FEP) patients and a naturalistic movie stimulation, instead of the more common resting state approaches. Functional brain imaging studies within subjects experiencing a naturalistic stimulus, such as viewing a movie, can complement resting state studies as they provide an excellent assay of emotional, motivational, social and cognitive functions in a way that has strong validity for everyday demands of brain function^13,14^. Compared to resting state studies, naturalistic stimuli may help a subject to stay alert and reduce head movement, which are crucial for connectivity studies. Such stimuli enhance the test-retest reliability of fMRI studies, and have been used successfully in numerous clinical studies^15–17^. Even if the strengths of functional connections depend to some extent on the task used, the overall architecture of the functional connectivity is stable during rest and various tasks, and average connectivity strengths during rest resemble those averaged across multiple different tasks^18^.

We hypothesized that striatal functional connectivity with the midbrain differs between FEP patients and healthy controls. We then used the midbrain regions differing in striatal connectivity between groups as a new seed for comparison of connectivity between groups across the brain. Assuming psychosis-related mesostriatal pathways project from these midbrain regions, alteration of connectivity of these regions with other parts of the brain in FEP patients might reflect alteration in regulation of the mesostriatal pathways. Although correlational findings from fMRI connectivity cannot inform directly about causality or the origins of mesostriatal dysfunction, such findings might pinpoint candidate connections for further studies to answer the central question, “What causes the mesostriatal dopaminergic dysfunction in psychotic disorders?”. To further our understanding of the findings, we correlated connectivity strengths with antipsychotic medication dose and symptom severity and compared the findings with those acquired in patients at follow-up imaging approximately 1 year later (1.26 + /−0.35 years).

## Methods

### Subjects

We included 159 participants (97 patients, aged 18–40 years, 62 age matched controls) from the Helsinki Early Psychosis Study (HEPS), Helsinki University Hospital, Finland. Participants were recruited 2010–2016 and data were collected 2010–2018. In the control group (*n* = 62) at baseline, one subject was excluded due to a neurological abnormality and one subject was excluded due to fMRI image misalignment that we could not reliably correct. One subject was excluded from symptom correlation analyses due to interviewers not being able to obtain a reliable evaluation of symptom severity at baseline. This provided 60 baseline control subject datasets for our analyses. In the patient group (*n* = 97) at baseline, twelve had inadequate data acquisition at the time of scanning, three had significant neurological findings, three were excluded due to significant head motion and three subjects had fMRI image misalignment that we could not reliably correct. This yielded 76 baseline FEP patient datasets for our analyses, and 75 baseline FEP patients for symptom correlation analyses. For all follow-up subjects in the patient group (*n* = 49) and control group (*n* = 43) there were no fMRI data exclusions required. One subject’s follow-up clinical data was unavailable, thus one dataset was absent from clinical correlation analyses.

The detailed methods about data collection have been reported previously^19,20^. Briefly, the inclusion criterion for FEP patients was having psychotic symptoms lasting over 24 h, with scores of 4–7 points on the Unusual thought content or Hallucinations in the Brief Psychiatric Rating Scale Extended (BPRS-E)^21^. We excluded patients with previous psychotic episodes and subjects whose psychotic symptoms were clearly related to substance use or a general medical condition. DSM-IV diagnoses were based on the Research Version of the Structural Clinical Interview for DSM-IV (SCID)^22^, which was complemented with information from medical records of all lifetime mental health treatment contacts^23^. BPRS interviews were done by trained psychiatric nurses or psychologists. The structured BPRS assessment by the UCLA Department of Psychiatry and Biobehavioral Sciences was translated into Finnish and adapted as a semistructured interview, and the training was designed and organized as recommended in Ventura et al. (1993)^24^. The same interviewers were trained to use SCID. Training was organized by a senior psychiatrist (JS) who also reviewed all medical records from the patients and the interviews together with the interviewers. Final diagnostic assessment was made by JS using all available information from the interviews and medical records. The diagnoses reported here are based on the longest follow-up data available from the patient. Diagnostic assessments were done at baseline for control subjects and at 2 months follow-up for patients, then again at the one year follow-up. We complemented BPRS with three items^25^ during both clinical assessments. At baseline, symptom severity was assessed for the worst lifetime period and for the past week. There was one follow-up assessment at 2 months between the scans to document the initial treatment response. At 12 months, symptom severity was assessed for the past week and for the worst period after the 2-month follow-up measurement (i.e. during the previous 10 months). Chlorpromazine equivalent doses were calculated using the defined daily dose method^26^.

### Image acquisition

Functional magnetic resonance imaging was attempted at baseline for all subjects unless deemed to be at risk due their mental state. We conducted the fMRI recordings at the AMI Centre of Aalto Neuroimaging, Aalto University, Finland. Due to the prescheduled change of the AMI Centre scanner during the study baseline, 32 subjects (19 patients, 13 controls) were scanned using a GE Signa VH/i 3 T scanner (scanner 1) with a 16-channel head coil and 134 subjects (85 patients, 49 controls) using a Siemens Skyra 3 T scanner (scanner 2) with a 32-channel head coil. For both scanners, the parameters for functional blood-oxygenation-level-dependent (BOLD) imaging were 36 slices, slice thickness 4 mm, matrix size 64 × 64, echo time 30 ms, repetition time 1.8 s, flip angle 75°, and field of view 24 cm (voxel size therefore 3.75 × 3.75 × 4 mm). We included participants from both scanners in the same analyses because multisite fMRI studies have shown that a 10% increase in sample size will increase statistical power as opposed to using only single scanner data^27^. Slices were aligned according to the line connecting the anterior and posterior commissures. T1-weighted structural images with 1 mm^3^ isotropic voxels and T2-weighted structural images were also acquired, and a clinical neuroradiologist evaluated these scans for brain abnormalities. At follow-up, neuroimaging and clinical assessment were repeated around 12 months using scanner 2 and successfully completed for 52 FEP patients and 43 healthy control subjects.

### Stimuli

During fMRI, the participants were shown five scenes in sequence from the fantasy film Alice in Wonderland, directed by Tim Burton (Walt Disney Pictures, Burbank, CA, 2010; dubbed into Finnish). The total duration was 7 min and 20 s, equaling 245 echo-planar imaging volumes. This film is partly animated with human actors present in every scene. The scenes show Alice’s wedding followed by her trip through a rabbit hole to Wonderland and subsequent fantasy events (see^28^ for full details). Scenes were projected without breaks using Presentation software (Neurobehavioral Systems Inc., USA) to a semi-transparent screen, visible to the subject via a mirror placed on the head coil. The soundtrack was delivered to the subject via plastic tubes connected to earplugs. Foam pads were added in and around the head coil for noise cancellation, and the sound level was adjusted to be comfortable for each subject.

### Data pre-processing

**Fig. 1:**
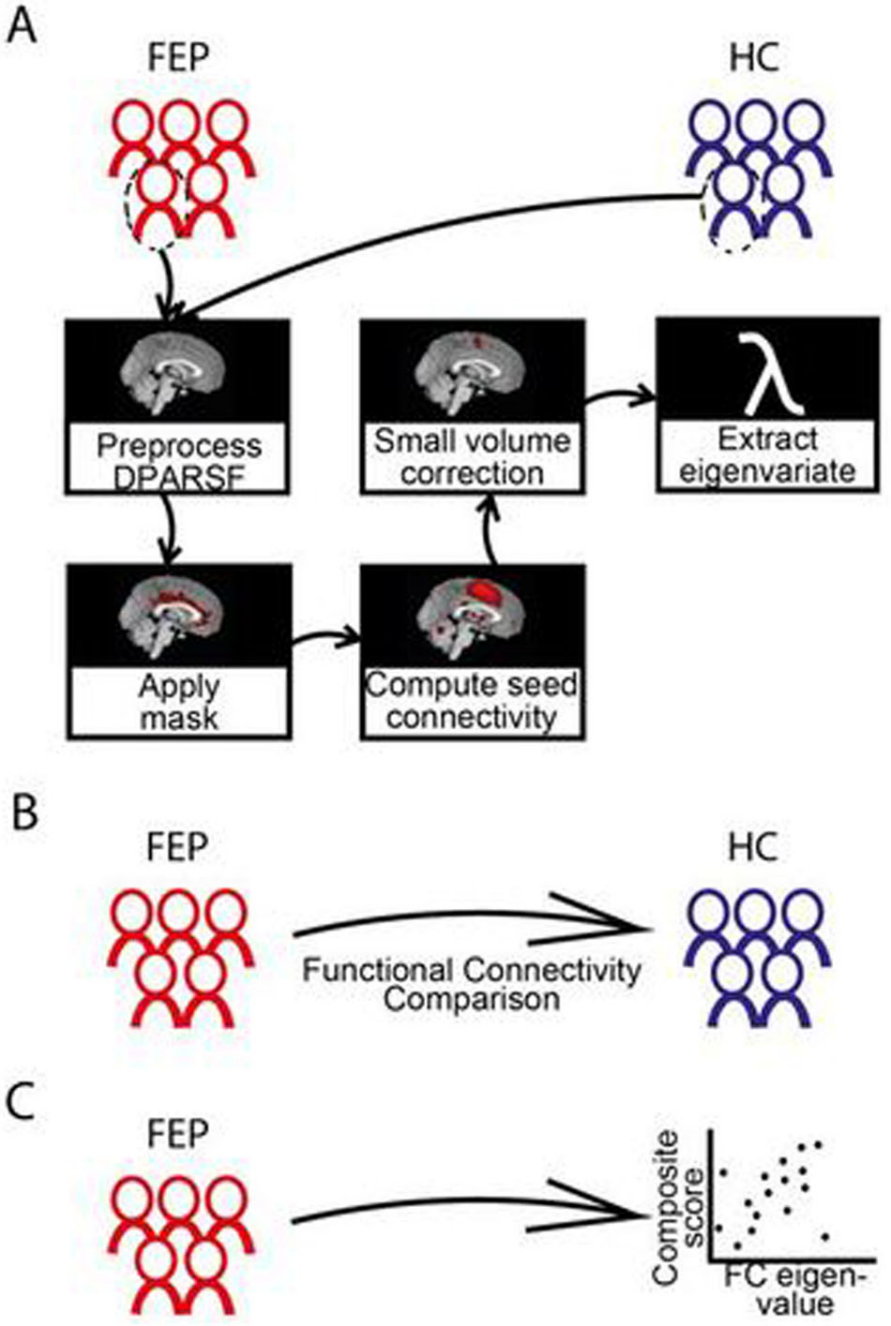
Diagram of the study design. **A** First fMRI connectivity analysis with striatal seeds. In the second connectivity analysis (not shown), the midbrain regions differing between groups were used as a new seed region. **B** The functional connectivity results generated by our seed-based approach were analysed by group level comparisons between FEP patients and healthy controls. **C** Functional connectivity measurements of FEP patients (FC eigenvalues) were correlated with antipsychotic drug dose equivalents and the severity of positive or negative symptoms composite scores at baseline and follow-up.

The fMRI data analysis steps are shown in Fig. 1. The fMRI data were pre-processed using DPARSF 5.0 software within DPABI^29^ run in MATLAB (Mathworks, Natick, USA). This included slice timing correction, realignment, co-registration to T1 images, and spatial normalization by DARTEL to the Montreal Neurological Institute (MNI) space. To reduce the effect of individual functional differences and to better fit the assumption of normal distribution, images were smoothed with an 8 mm full-width-at-half-maximum Gaussian kernel. We used framewise displacement (FD)^30^ to calculate a score for movement for each time point (1 time point = 1 repetition time) and a mean score across all time points, excluding subjects with mean FD Jenkinson > 0.2 mm or percent of FD Power > 0.5 mm > 0.2 (i.e., 20%) from further analyses.

### Functional connectivity methodology

**Fig. 2:**
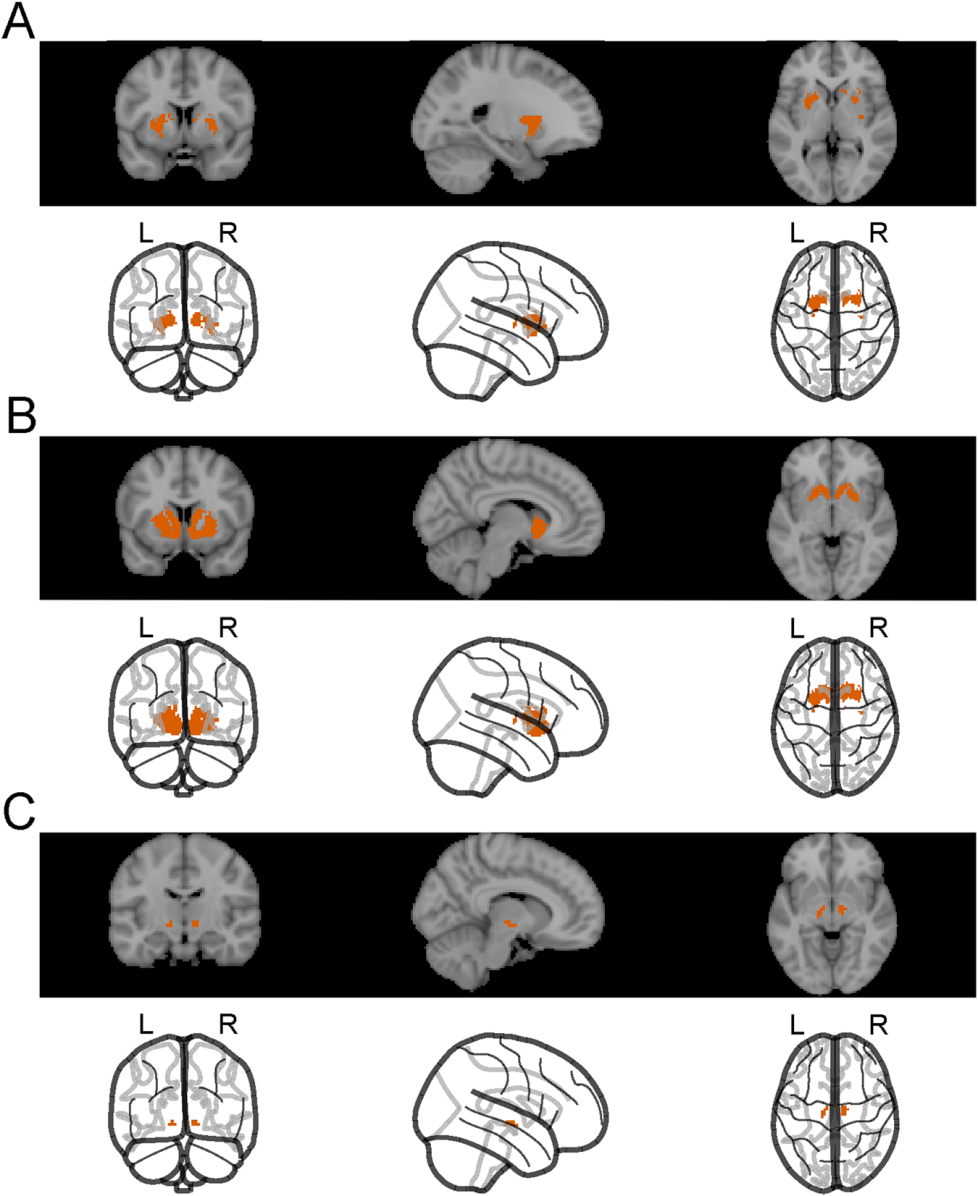
The seed regions. **A** Seed 1. A dorsal striatal seed mask based on NeuroSynth ‘motivational’ output subtracted from the NeuroSynth ‘associative’ output. **B** Seed 2. A ventral striatal seed mask created by subtracting the ‘associative’ output from the ‘motivational’ output. **C** Seed 3. Our second analysis utilised the midbrain regions carrying significant hypoconnectivity with the dorsal striatum (Seed 1) as a new seed, Seed 3.

After pre-processing, DPARSF 5.0 was used to compute functional connectivity with a seed-based approach. Firstly, two striatal seed masks were created using the NeuroSynth database (neurosynth.org) by search terms ‘associative’ and ‘motivational’ with respective.nii file outputs generated. This approach intends to create seed regions defined by striatal functional activity rather than its precise anatomical borders. To generate a representative dorsal associative striatal seed (Seed 1, Fig. 2A), the motivational output was subtracted from the associative output in SPM12. To generate a representative ventral striatal seed mask (Seed 2, Fig. 2B), the associative output was subtracted from the motivational output.

The functional connectivity images generated by our seed-based approach were compared between FEP patients and healthy control subjects in a longitudinal analysis using MRM toolbox^31^ and in two-sample t-tests in SPM12, both with and without adjustment for scanner type. We used region of interest analysis within one midbrain anatomical mask (WFU PickAtlas, RRID:SCR_007378). All analyses were corrected for multiple comparisons at the cluster level with primary voxel-level threshold *p* = 0.001. The resulting significant midbrain clusters were used for subsequent mask-based eigenvariate extraction of connectivity values for each FEP subject, to quantify striato-midbrain connectivity. These values were extracted for both baseline data and follow-up data, with the same masks used upon baseline data and follow-up data, to allow us to make regional FC comparisons over time as a post hoc analysis. Our second analysis utilized the midbrain clusters that differed in connectivity with the dorsal striatum (Seed 1) between groups as a new midbrain seed (Seed 3, Fig. 2C). This approach aimed to utilize localization of the functionally defined midbrain alterations to further assess midbrain-related circuitries of altered connectivity. To estimate validity of the midbrain connectivity findings, we compared them with average midbrain connectivity of 1000 subjects in the Neurosynth database.

### Post hoc analysis on extracted FC values

Extracted FC values were normally distributed (Shapiro-Wilk test) and tested for Pearson’s partial correlations with positive and negative symptom composite scores. These correlations were controlled for scanner type and antipsychotic dose as antipsychotics may affect striatal connectivity^32^. FC values between time points and scanner groups were compared using paired and unpaired samples Student’s t-tests, respectively. These methods were completed in SPSS Statistics (Version 27, IBM, New York). For FEP patients, evaluation of FC changes over time in correlation with negative or positive symptoms was achieved using repeated measures correlation analysis (rmcorr)^33^ in R Studio (rstudio.com, Boston, MA). This method relies on repeated measures of FC within each subject and specifically tests for a common association with other variables within each subject. It then provides a correlation plot over time for the FEP group. Unlike ANOVA, potentially confounding variables between subjects such as sex, age and BMI have no influence on the rmcorr correlation analysis.

To test the hypothesis that functional connectivity was associated with the severity of positive and negative symptoms, we calculated two positive symptom and two negative symptom composite sum scores with BPRS-E^21^ and SANS^25^ items as follows: Negative 1; BPRS 16 (blunted affect) + SANS global rating for Anhedonia/Asociality + SANS global rating for Alogia + SANS global rating for Avolition/Apathy. Negative 2; BPRS (16 (blunted affect) + 17 (emotional withdrawal) + 18 (motor retardation)) x 0.33. Positive 1 and 2; past week (Positive 1) and worst ever episode (Positive 2) sum of BPRS 10 (hallucinations) + 11 (unusual thought content, i.e. delusions) + 12 (bizarre behaviour) + 15 (conceptual disorganization, i.e. positive formal thought disorder).

### Ethical standards

All procedures contributing to this work comply with the ethical standards of the Finnish national and institutional committees on human experimentation, and with the Helsinki Declaration of 1975, revised in 2008. The study protocol was approved by the Ethics Committee of the Hospital District of Helsinki and Uusimaa (diary numbers 257/12/03/03/2009 and 226/13/03/03/2013), and written informed consent was obtained from each subject before participation.

## Results

**Fig. 3:**
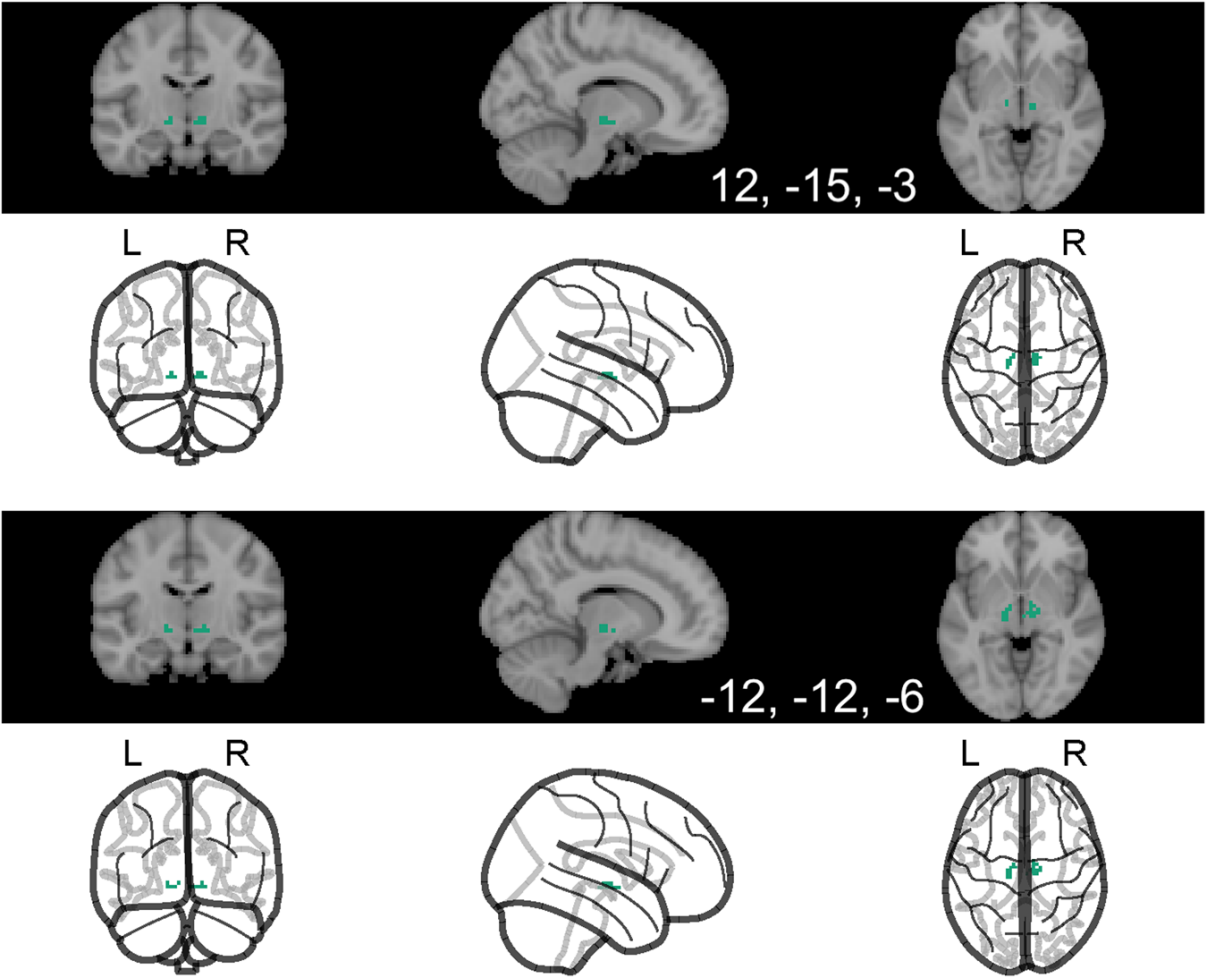
Functional hypoconnectivity between the dorsal striatum and the midbrain. Images of the baseline clusters of significant functional hypoconnectivity of the midbrain with Seed 1 (dorsal striatum, Fig. 2A). There were no clusters of midbrain hypoconnectivity with Seed 2 (ventral striatum, Fig. 2B). All findings were made by groupwise comparisons between FEP patients (*n* = 76) and healthy matched controls (*n* = 60) at baseline. No significant connectivity changes between these regions were seen at follow-up 12 months later. Primary voxel *p*-value of 0.001 was used in image and in cluster level statistics.

Sample characteristics are shown in Table 1. First episode psychosis (FEP) patients and control subjects did not differ regarding age or sex either at baseline or follow up. Our movie-based fMRI identified dorsal striatal functional hypoconnectivity with the midbrain at baseline presentation of FEP, as compared with controls (Table 2 and Fig. 3). Our repeated measures longitudinal analysis identified no significant FC changes over time, either corrected or uncorrected for scanner type (*p* > 0.05). Therefore, masks of the voxels of significantly reduced FC found at baseline were used to extract equivalently located FC values from follow-up data for FC comparisons only as a post hoc analysis.

**Table 1:** Sample characteristics at baseline and follow-up.

Baseline	Control subjects^a^ *N* = 60	FEP patients^a^ *N* = 76	Group Difference^b^
Male	38 (63.3)	51 (67.10)	*p* = 0.686
Age	23.97 (19.10–43.87, 6.83)	23.98 (18.26–39.06, 6.55)	*p* = 0.420
Years of education	15.00 (11.00–22.00, 3.50)	12.50 (9.00–22.00, 3.88)	*p* = 0.004
BPRS 10	1 (1–1, 0.00)	2 (1–6, 4.00) (*N* = 75)	***p*** < **0.001**
BPRS 11	1 (1–2, 0.00)	4 (1–7, 5.00) (*N* = 75)	***p*** < **0.001**
BPRS 12	1 (1–1, 0.00)	1 (1–6, 1.00) (*N* = 75)	***p*** < **0.001**
BPRS 15	1 (1–2, 0.00)	1 (1–5, 0.00) (*N* = 75)	***p*** = **0.003**
BPRS 16	1 (1–3, 0.00)	1 (1–5, 2.00) (*N* = 75)	***p*** < **0.001**
SANS 1	0.00 (0.00–1.00, 0.00)	0.00 (0.00–3.00, 1.00) (*N* = 75)	***p*** < **0.001**
SANS 2	0.00 (0.00–1.00, 0.00)	2.00 (0.00–5.00, 3.00) (*N* = 75)	***p*** < **0.001**
SANS 3	0.00 (0.00–2.00, 0.00)	3.00 (0.00–5.00, 2.00) (*N* = 75)	***p*** < **0.001**
Antipsychotic dose	0.00 (0.00–0.00, 0.00)	300.00 (0.00–1320.00, 300.00)	***p*** < **0.001**
GAF	85.00 (55–95, 14.25)	37.50 (15–75, 8.00)	***p*** < **0.001**
DSM-IV diagnosis	296.25/296.26/296.32/296.36 Major depressive disorder (*n* = 10)	292.12 Drug induced psychotic episode, cannabis (*N* = 1)	
	300.21 Panic disorder with agoraphobia (*n* = 1)	295.3 Paranoid schizophrenia (*n* = 12)	
	300.23 Social phobia (*n* = 1)	295.4 Schizophreniform disorder (*n* = 18)	
	300.3 Obsessive-compulsive disorder (*n* = 1)	295.7 Schizoaffective disorder (*n* = 1)	
	305 Alcohol abuse (*n* = 1)	295.9 Schizophrenia undefined (*n* = 15)	
	307.1 Anorexia Nervosa (*n* = 1)	296.04/296.44 Bipolar type 1 disorder (*n* = 7)	
	311 Depressive disorder NOS (*n* = 1)	296.24/296.34 Major depressive affective disorder with psychotic features (*n* = 4)	
		298.8 Brief psychotic disorder with psychotic features (*n* = 2)	
		298.9 Unspecified psychosis (*n* = 15)	
		300 OCD (*n* = 1)	

Follow-up	Control subjects^a^ *N* = 43	FEP patients^a^ *N* = 48	Group Difference^b^
Male	32 (74.40)	29 (60.40)	*p* = 0.179
Age	25.24 (21.07–41.50, 6.63)	25.16 (19.41–36.93, 5.70)	*p* = 0.382
BPRS 10	1 (1–2, 0.00)	2 (1–6, 0.00)	*p* = 0.070
BPRS 11	1 (1–2, 0.00)	4 (1–7, 1.00)	***p*** < **0.001**
BPRS 12	1 (1–1, 0.00)	1 (1–4, 0.00)	*p* = 0.057
BPRS 15	1 (1–2, 0.00)	1 (1–4, 0.00)	*p* = 0.214
BPRS 16	1 (1–2, 0.00)	1 (1–4, 1.00)	***p*** = **0.002**
SANS 1	0.00 (0.00–0.00, 0.00)	0.00 (0.00–3.00, 0.00)	***p*** < **0.006**
SANS 2	0.00 (0.00–2.00, 0.00)	1.00 (0.00–4.00, 2.00)	***p*** < **0.001**
SANS 3	0.00 (0.00–2.00, 0.00)	3.00 (0.00–4.00, 3.00)	***p*** < **0.001**
Antipsychotic dose	0.00 (0.00–60.00, 0.00)	190.00 (0.00–1050.00, 273.75)	***p*** < **0.001**
GAF	85.00 (40–96, 11.25)	50.00 (30–90, 28.00)	***p*** < **0.001**

^a^Frequency (%) or median (range, IQR). ^b^Mann-Whitney *U* test or Pearson Chi-square test. Significant *p*-values shown in bold. *FEP* First-episode psychosis, *BPRS* Brief Psychiatric Rating Scale, *SANS* Scale for the Assessment of Negative Symptoms, *BPRS 10* Hallucinations item, *BPRS 11* Unusual thought content, *BPRS 12* Bizarre behavior, *BPRS 15* Conceptual disorganization, *BPRS 16* Blunted affect, *SANS 1* Alogia, *SANS 2* Avolition, *SANS 3 A*nhedonia, *GAF* Global assessment of functioning.

**Table 2:** Summary of main functional connectivity findings by groupwise comparisons at baseline. Regional functional connectivity (FC) differences were not seen at 12 months follow-up. Clusters from the hypoconnected regions in first episode psychosis patients at baseline are shown together in Fig. 6.

Seed	Connected region x,y,z coordinates	*P*-value (scanner corrected)	*K* (number of voxels)	Functional connectivity (FC) at baseline	Functional connectivity associations
Dorsal striatum	Midbrain	0.000003*	13	Decreased in patients	Baseline antipsychotic dose negative correlation
			10		
					(*r* = −0.289, *p* = 0.012)
		0.00006*			
	12, −15, −3				
	−12, −12, −6				
Ventral striatum	Midbrain			No change	N/A
Midbrain	Cerebellum	0.007**	17	Decreased in patients	N/A
	18, −60, −30				
	−39, −42, −45	0.007**	17		

^*^Corrected for multiple comparisons within region of interest; **Corrected for multiple comparisons within the whole brain.

**Fig. 4:**
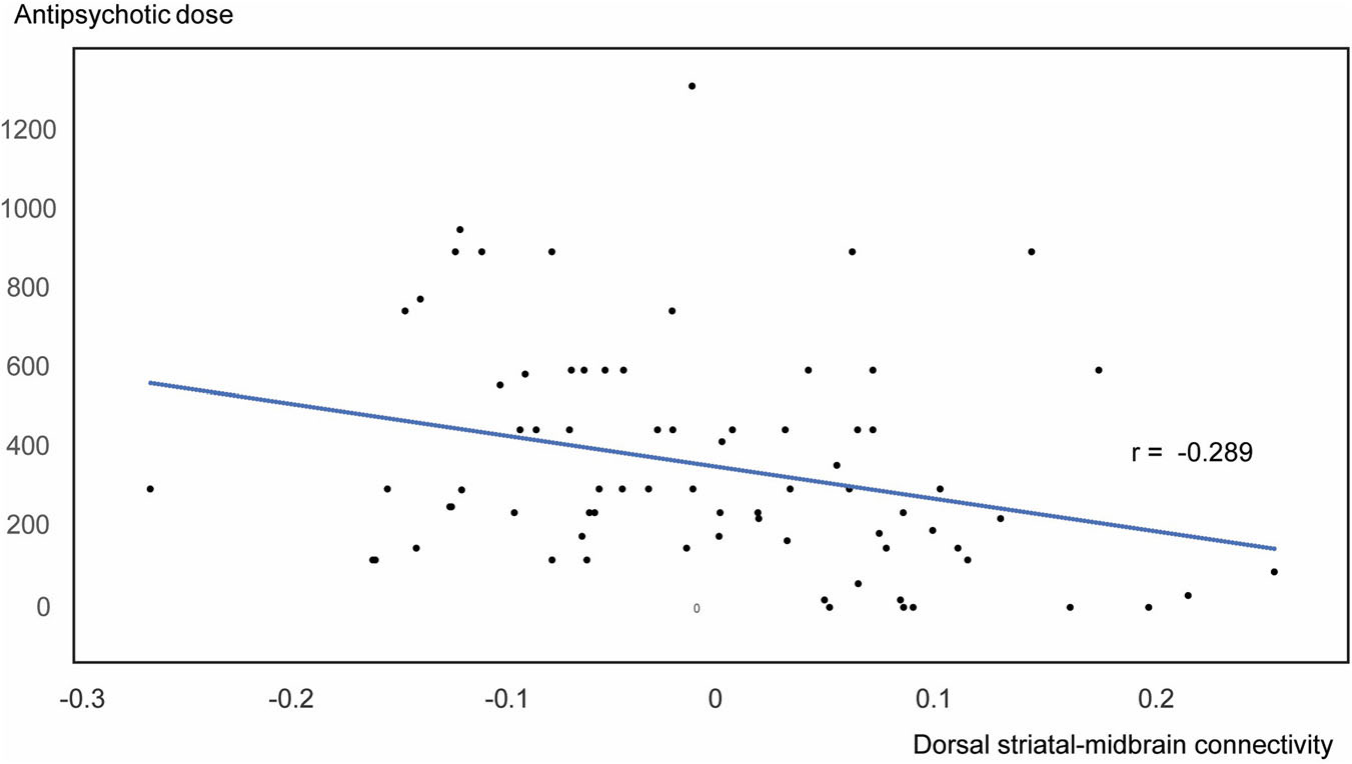
Functional connectivity relationship with baseline antipsychotic drug dose. Scatter plot demonstrating partial correlation between baseline dorsal striatal-midbrain functional connectivity and baseline antipsychotic medication dose, controlled for scanner type.

Clusters of hypoconnectivity of the striatum with the midbrain baseline are shown in Fig. 3. The midbrain cluster coordinates did not change when comparisons were corrected for scanner type at baseline. Specifically, reduced baseline FC was only seen with our dorsal (associative) striatal seed (Seed 1, Fig. 2A), and not with our ventral (motivational) striatal seed (Seed 2, Fig. 2B). Controlling for scanner type, dorsal striato-midbrain FC was negatively correlated with antipsychotic drug dose equivalents at baseline only (*r* = −0.289, *p* = 0.012, Fig. 4). There were no partial correlations of baseline FC with positive or negative symptoms (*p* > 0.05). In FEP patients, inter-regional functional connectivity was not significantly different between baseline and follow-up (longitudinal analyses and paired samples Student’s t-tests, *p* > 0.05). When evaluating correlations between functional connectivity and negative symptom, positive symptom, or antipsychotic dose changes over time, the rmcorr approach yielded no correlations over the duration between baseline and follow-up (*p* > 0.05).

**Fig. 5:**
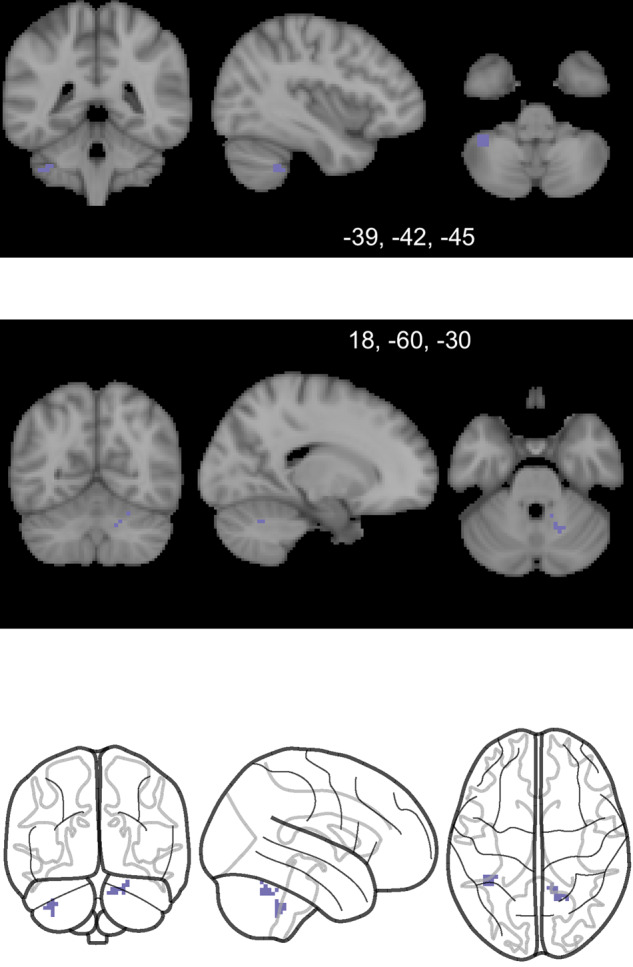
Functional hypoconnectivity between the midbrain and the cerebellum. Images of the clusters of significant cerebellar functional hypoconnectivity with the midbrain (Seed 3, Fig. 2C). The greyscale images show two representative slice locations, which are both compiled in the line diagram row. Primary voxel p value of 0.001 was used in image and in cluster level statistics.

Groupwise, functional connectivity between the midbrain seed (Seed 3) and areas of the cerebellum was reduced at baseline in patients when compared with healthy controls (Fig. 5). Connectivity of 1000 subjects in Neurosynth database suggested these cerebellar regions to be connected with the midbrain seeds in healthy subjects (*r* = 0.04–0.07) supporting validity of these connections. The cerebellum showed no significant groupwise FC changes with the midbrain at follow-up 12 months later (*p* > 0.05). In FEP patients, midbrain-cerebellar functional connectivity was not significantly different between baseline and follow-up (longitudinal analyses and paired samples Student’s t-test, *p* > 0.05). Controlling for scanner type, midbrain-cerebellar FC did not correlate with antipsychotic drug dose equivalents at baseline or follow-up (*p* > 0.05). The midbrain-cerebellar FC did not correlate with negative symptoms or antipsychotic dose at baseline or follow-up (*p* > 0.05). As with striatal FC, the rmcorr approach showed no significant correlations between midbrain-cerebellum FC and either positive symptom, negative symptom, or antipsychotic dose changes over the 12 months duration from baseline to follow-up (*p* > 0.05).

**Fig. 6:**
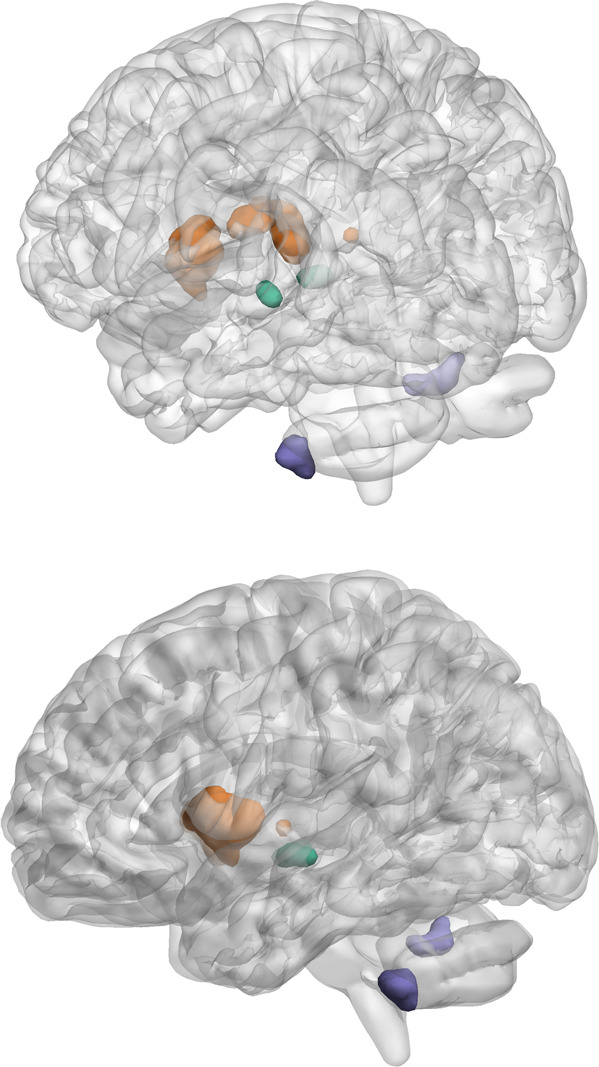
A whole brain representation of the FEP functional hypoconnectivity network seen through groupwise comparisons with healthy controls at baseline. The three linked regions include clusters within the dorsal striatum (Seed 1, orange), the midbrain (Seed 3, green) and the cerebellum (purple). The whole brain surface representation is created by a previously published method in BrainNet Viewer^27^. Primary voxel *p*-value of 0.001 was used in image and in cluster level statistics.

Taken together, our results show groupwise functional hypoconnectivity between the dorsal striatum (Seed 1, Fig. 2A) and midbrain regions (Fig. 3A), and between those same midbrain regions and areas of the cerebellum (Fig. 5). This three-point network of hypoconnectivity found in our FEP study at baseline is illustrated in Fig. 6. The main findings and their correlations are summarized in Table 2.

## Discussion

Our research identified dorsal striatal hypoconnectivity with the midbrain during naturalistic stimulus processing at the time of FEP presentation (baseline). Furthermore, the midbrain regions that showed reduced connectivity with the dorsal striatum showed hypoconnectivity with the cerebellum, which is suggested to be involved in regulation of the mesostriatocortical dopaminergic pathways^34^. Mesostriatal and meso-cerebellar hypoconnectivity were not seen at follow-up.

We focused on the connectivity of the mesostriatal interactions that have now become highly implicated in psychosis^35^. Aberrant striatal connectivity has been well documented and reviewed^2^. In line with our findings, dopaminergic dysfunction has most frequently been seen in the dorsal (associative) striatum^36–38^. Supporting this, fMRI studies have shown dorsal striatal hypoconnectivity with cortical networks in schizophrenia^39–43^ and in patients with clinical high risk of psychosis^44^. Midbrain connectivity is of special interest as dopaminergic activity characterized by increased synthesis and release of dopamine in psychotic disorders originate in the midbrain^7,8^.

Mesostriatal connectivity has previously been positively associated with positive symptoms^40^ and negatively with negative symptoms^45^. Furthermore, functional striatal resting state abnormalities that can predict schizophrenia severity do include hypoconnectivity between the striatum and the midbrain^6^. Our findings extend these lines of evidence by suggesting mesostriatal hypoconnectivity prevails during naturalistic stimulus processing in FEP, in association with medication dose. No significant individual or repeated measures functional connectivity correlations with positive symptoms were found. We also observed lower baseline dorsal striato-midbrain connectivity in association with higher baseline antipsychotic drug dose equivalents in FEP. In line with this, fMRI before antipsychotic treatment^46,47^ as well as during treatment^48^ of early schizophrenia has shown decreased midbrain connectivity with the dorsal striatum during the resting state. The present study did not find associations between antipsychotic use and longitudinal functional connectivity changes, but interestingly, antipsychotic dose correlated negatively with meso-striatal connectivity at the baseline. This finding may reflect blocking of mesostriatal interactions during antipsychotic treatment and supports the assumption that the present fMRI findings may reflect functioning of the mesostriatal dopaminergic activity.

Little is known about midbrain connectivity beyond the striatum^7^. Therefore, it is interesting that the midbrain regions showing group difference in their striatal connectivity also differed between groups in their connectivity with the cerebellum. The cerebellum is known to be important in cognitive and emotional-motivational circuitries beyond its motor functions. It was involved in the influential hypothesis of cognitive dysmetria of Andreasen and coworkers^49^, and further evidence has supported the role of the cerebellum in schizophrenia^34^. Structural connections are known to exist at least between the fastigial cerebellar nuclei and the midbrain, and stimulation of the fastigial and dentate nuclei alters dopamine functioning in the medial prefrontal cortex^34^. In line with this, the present midbrain and cerebellar regions were connected in a large sample of healthy subjects in Neurosyth fMRI database. The present findings support further studies to assess the potential role of the cerebellum in regulation of mesostriatocortical dopaminergic activity in psychosis. Such studies could have important therapeutic implications regarding, for example, targeting transcranial magnetic stimulation of the cerebellum^34^.

### Study considerations and future research

The reduction in dorsal striato-midbrain-cerebellar connectivity were characteristic for FEP patients during the baseline study when symptoms were, on average, more severe. Larger longitudinal studies are needed to assess whether such changes could represent a state-like dysfunction in connectivity that underpin such symptoms.

The present study used naturalistic stimuli in lieu of resting state data, and thus we cannot directly compare these two methods of obtaining fMRI data. It is possible that the present movie stimulus elicits different connectivity of the midbrain with the striatum and cerebellum than the resting state. Further research would ideally contain data obtained both at rest and during naturalistic stimuli, to assess the relationships between changes occurring in each state. Furthermore, analyses of dynamic connectivity during a task can be used to link task characteristics to changes in connections of interest. Importantly, naturalistic stimuli may help to reduces the likelihood of possible sources of error related to sleepiness and head movement, uncontrollability of psychological state during imaging and the validity of the stimulus for everyday life (fixation cross versus a meaningful movie).

We defined midbrain seeds based on their functional connectivity in the present sample, which may enhance validity of seed regions for the study questions. The resulting midbrain seeds were small (altogether 621 mm^2^), comparing to only about 11 original voxels in image acquisition. Considering the accuracy of DARTEL normalization^50^ and smoothing the data to compensate for interindividual differences, the seed size appears sufficient.

In summary, the present study supports continued clinical and research attention upon functional hypoconnectivity within dorsal striatal dopaminergic interactions and associated cerebellar activity in early psychosis. Robust evaluation of striatal functional connectivity abnormalities within the mesostriatal system especially^6^ with the added context of patient risk factors may enhance our predictive models for psychosis severity when employed as it first presents. Our results provide novel insight on functional connectivity changes that could eventually help to access causal explanation for positive and negative symptoms in psychotic disorders.

## Data Availability

To protect vulnerable study subjects, data of the present study is not publicly available. Inquiries about the data can be addressed to the corresponding author.
